# Bordetella Pertussis in Children: A Retrospective Analysis of the Clinical Impact and the Role of Vaccination

**DOI:** 10.3390/life15101514

**Published:** 2025-09-25

**Authors:** Elena-Roxana Matache (Vasilache), Gabriela Gurau, Valerian-Ionut Stoian, Andreea Eliza Zaharia, Manuela Ciocoiu, Nicoleta-Maricica Maftei, Paula Constantinide, Madalina Nicoleta Matei, Aurel Nechita, Dana Tutunaru

**Affiliations:** 1 Department of Morphological and Functional Sciences, Faculty of Medicine and Pharmacy, “Dunarea de Jos” University, 800008 Galati, Romania; elena.matache@ugal.ro (E.-R.M.); gabriela.gurau@ugal.ro (G.G.); 2 Medical Laboratory Department, “Sf. Ioan” Emergency Clinical Hospital for Children, 800487 Galati, Romania; nicoleta.aron@ugal.ro; 3 Center for Research and Technology Transfer in the Medico-Pharmaceutical Field, “Dunărea de Jos” University, 800008 Galaţi, Romania; paula.constantinide@ugal.ro (P.C.); aurel.nechita@ugal.ro (A.N.); dana.tutunaru@ugal.ro (D.T.); 4 Medical Department, Faculty of Medicine and Pharmacy, “Dunarea de Jos” University, 800008 Galati, Romania; 5 National Institute for Public Health, 050463 Bucharest, Romania; 6 Department of Morpho-Functional Sciences II, “Grigore T. Popa” University of Medicine and Pharmacy, 700115 Iasi, Romania; manuela.ciocoiu@umfiasi.ro; 7 Department of Pharmaceutical Sciences, Faculty of Medicine and Pharmacy, “Dunarea de Jos” University, 80008 Galati, Romania; 8 Department of Dental Medicine, Faculty of Medicine and Pharmacy, “Dunarea de Jos” University, 800008 Galati, Romania; 9 Clinical Medical Department, Faculty of Medicine and Pharmacy, “Dunarea de Jos” University, 800008 Galati, Romania; 10 Medical Laboratory Department, “Sf. Apostol Andrei” Emergency Clinical Hospital, 800578 Galati, Romania

**Keywords:** *Bordetella pertussis*, RT-PCR, vaccination, co-infection, rhinovirus, children

## Abstract

Pertussis, a highly contagious disease, contributes to a great number of hospitalizations among children, with an increased risk of morbidity and mortality. The aim of the study was to investigate the epidemiological and clinical features of *B. pertussis* infections among hospitalized children and to compare the clinical course according to vaccination status and the presence of co-infections. We performed a retrospective study, which included patients positive for *B. pertussis* detected by multiplex RT-PCR panels, from September 2022 to May 2025. Out of 2493 samples, 84 tested positive for *B. pertussis* (3.37%). Age-appropriate immunization was achieved in 19.1% (16/84) cases, 10.7% (9/84) were incompletely vaccinated, 9.5% (8/84) did not meet the age criteria and 60.7% (51/84) were not vaccinated. Infants ≤ 3 months were more susceptible to mixed co-infections (52%), had a more severe course, with transfers to the ICU (32%) and a prolonged average length of stay (9.2 days). Co-infections were found in 39.3% cases, rhinovirus being the most common agent (17.9%). *B. pertussis* and rhinovirus co-infection was associated with a decreased SpO_2_ level (<92%) and increased CRP and Ferritin levels.

## 1. Introduction

*Bordetella pertussis*, a Gram-negative coccobacillus, causes whooping cough, a highly contagious disease that, despite vaccination strategies, continues to affect all age groups [1]. The disease manifests itself with paroxysmal coughing episodes, lasting up to several months and causing severe forms, especially amongst infants under 6 months. Infants too young to be vaccinated are at high risk of complications, such as encephalitis or pneumonia, which can be fatal [2,3]. Frequently, they are initially treated for acute bronchitis or bronchiolitis, presenting with atypical symptoms, including apnea, cyanosis, wheezing, and nonspecific laboratory tests [1,4].

Disease prevention and control through immunization (vaccination) remains the most effective method [5]. Up until now, two types of vaccines have been developed: whole-cell pertussis (wP), obtained from inactivated pathogens, and the acellular pertussis vaccine, which contains inactivated pertussis toxin and other purified bacterial antigens, such as filamentous hemagglutinin, pertactin, and fimbriae [4]. In terms of efficiency, whole-cell pertussis vaccine is the most effective in preventing transmission [1,6], whereas the acellular vaccine has fewer adverse reactions. The latter was implemented in Romania in 2008, and the pertussis vaccination schedule in 2015 comprises three doses of hexavalent DTPa-IPV-HB-Hib. This schedule consists of multiple doses administered at ages 2, 4 and 11 months, followed by a first booster with the same type of vaccine at age of 5–6 years, and another booster at the age of 14 years, with dTpa for adults [7]. Unlike it is the case with other infectious and contagious diseases, a person can become infected with *Bordetella pertussis* several times during their lifetime, because the antibodies that are developed after infection or after immunization do not persist in time [8].

Although the efficacy of vaccines has been demonstrated to reduce the burden of disease by 90%, a factor that ought to drive vaccination coverage close to 100%, the WHO estimated globally a percentage of just 84% infants (108 million) who received the complete vaccination schedule in the first year of life in 2023 [9,10]. There were no major differences in 2024, where the organization estimated a global full vaccination rate of 85% [9]. Regarding unvaccinated children, their number globally surged from 13.9 million in 2022 to 14.5 million in 2023, and 14.3 million in 2024 [10,11].

The most recent reported data show a significant increase in the incidence of *B. pertussis* cases in 2023 compared to the preceding year in several European countries, such as Denmark (6059 vs. 52 cases), Spain (2560 vs. 215 cases), Austria (2790 vs. 164 cases), Norway (1201 vs. 44 cases), Belgium (1046 vs. 80 cases), the UK (942 vs. 79 cases), Belarus (881 vs. 28 cases), and Ukraine (707 vs. 32 cases) [12,13]. As for Romania, after peaking in 2019, with a relatively moderate incidence of 110 cases, a remarkable decrease was registered during following years, from 18 cases in 2020 to a single case reported in 2021 [14]. Moreover, 16 cases were reported in 2023, with a difference of seven more cases than in 2022, and with a three-dose vaccination coverage rate during the first year of life of only 56.1% [10,11]. The year 2024 was marked by a massive increase, reaching 2862 reported cases [12,13,15].

The PCR test, performed from a pharyngeal swab or nasopharyngeal aspirate, remains the gold standard for diagnosing *B. pertussis*, characterized by high sensitivity (90.7–95%) and specificity (93–100%) [16].

Recent studies have shown an increased frequency of viral co-infections among infants with *B. pertussis*, reaching one third of cases [17]. However, the association of mixed co-infections with disease severity is unclear [18].

The aim of this study was to analyze epidemiological and clinical characteristics in patients diagnosed with *B. pertussis*. We also aimed to investigate the clinical severity in patients with only *B. pertussis* compared to those with co-infections, taking into consideration the role of vaccination in these patients.

## 2. Materials and Methods

### 2.1. Study Design and Configuration

This retrospective study was conducted within the “Sf. Ioan” Children’s Emergency Clinical Hospital in Galati, Romania, between September 2022 and May 2025, on a group of 2493 patients. The study was approved by the Ethics Committee of the “Sf. Ioan” Children’s Emergency Clinical Hospital (No 12107, on 12 June 2025). Specific informed consent was not included in this analysis, since we have collected data from standard care procedures, without requiring additional interventions on patients.

### 2.2. Patient’s Enrolment

Inclusion criteria:

- children and adolescents aged 0–18 years
- hospitalized patients with symptoms suggestive of respiratory infection
- patients undergoing the Allplex™ Respiratory Panel Assay kit 4 (Seoul, Republic of Korea)

Exclusion criteria:

- chronic heart or lung diseases and immunodeficiency diseases

### 2.3. Data and Sample Collection

Demographic data, symptomatology, laboratory test results, chest imaging, length of hospital stay and treatment details were collected from the hospital computer system. Vaccination status data were obtained from the National Electronic Vaccination Registry.

Nasopharyngeal exudates were collected from each patient using synthetic fiber swabs with plastic rods, which were placed in sterile tubes with 3 mL of universal transport medium (UTM). Samples were processed immediately or frozen at −70 degrees until processing.

### 2.4. Multiplex RT-PCR Analysis

Identification of *B. pertussis* was performed with the Allplex™ Respiratory Panel Assays kit 4 (Seegene Inc., Seoul, Republic of Korea), a kit that simultaneously detects six other bacteria: *Streptococcus pneumoniae*, *Haemophilus influenzae*, *Legionella pneumophila*, *Mycoplasma pneumoniae*, *Chlamydophila pneumoniae* and *Bordetella parapertussis*. Co-detection of viral pathogens was also possible, most patients being tested concurrently with other screening panels—Respiratory Panel Assays kits 1,2,3 (Seegene Inc., Republic of Korea) for 15 viral respiratory agents: human metapneumovirus, human rhinovirus A/B/C, human adenovirus, human enterovirus, human bocavirus 1/2/3/4, human parainfluenza 1/2/3/4, human coronaviruses (229E, NL63, OC43), SARS-CoV-2, RSV and Influenza A/B. Nucleic acids were extracted using the STARMag 96 × 4 universal kit (Seegene Inc., Republic of Korea) with a Nimbus automated extractor (Seegene Inc., Republic of Korea), and qRT-PCR was performed with a CFX96 amplifier (Bio-Rad, Hercules, CA, USA).

### 2.5. Statistical Analysis

Data were collected and analyzed using the IBM SPSS Statistics software (version 26). We used the χ^2^ test and Fisher’s exact test for categorical variables, and the Kruskal–Wallis test for continuous variables. The significance level was set at <0.05.

## 3. Results

**Figure 1:**
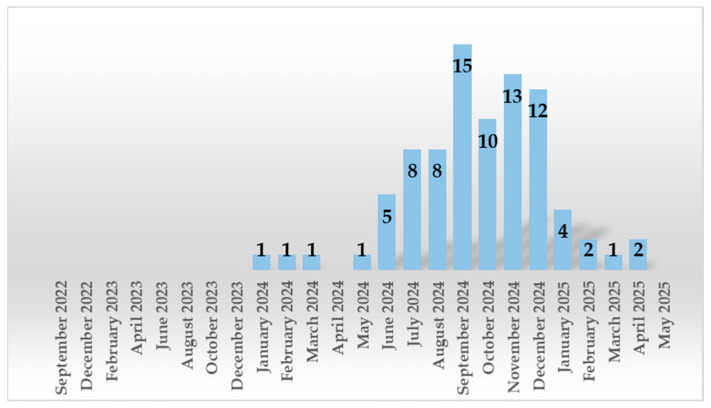
Monthly and yearly distribution of *B. pertussis* positive cases.

Out of 2493 nasopharyngeal exudates collected, 3.37% (*n* = 84) tested positive for *B. pertussis*. The distribution of positive cases by year was as follows: 0% (0/183) in 2022, 0% (0/709) in 2023, 7.12% (75/1053) in 2024, and 1.64% (9/548) by May 2025. A significant increase in *B. pertussis* infections was observed starting in June 2024, reaching a peak in September 2024 (15.8%) (Table 1 and Figure 1).

*B. pertussis* infections predominated in girls, with a percentage of 56%. Most cases were detected in children aged 13–72 months (32.1%), followed by patients aged 0–3 months (29.8%). Out of 84 positive patients, 25 were vaccinated against *B. pertussis* (29.8%), of which 16 (19.1%) received age-appropriate doses, and 51 children (60.7%) were not vaccinated at all (Table 2).

Reported symptoms were analyzed in patients of different age groups. We observed that dyspnea and cyanosis predominated among *B. pertussis* patients ≤ 3 months, with a percentage of 68% and 28%, respectively (*p* = 0.001; *p* = 0.002). Moreover, this group of patients was more susceptible to mixed co-infections, with a rate of 52% (*p* = 0.029), and had prolonged hospitalization, with a mean of 9.2 days (*p* = 0.022). Post-cough vomiting occurred in 33.3% of the cases in children aged 13–72 months (*p* = 0.045) (Table 3).

Regarding the disease progression according to age, 32% (*n* = 8) of *B. pertussis* patients ≤ 3 months were transferred to the ICU (*p* = <0.001) and only one required mechanical ventilation, presenting cardiorespiratory arrest. No deaths were recorded (Table 4).

Out of 84 *B. pertussis* positive patients, 63 (75%) were admitted with symptoms suggestive of lower respiratory tract infection (bronchitis, pneumonia). The mean length of the hospital stay was 7 (2–20) days, with 35.7% (30/84) of patients exceeding this mean. Regarding the severity of infection, only six patients (7.1%) developed respiratory failure, while 78 children (92.9%) had a milder form.

Single *B. pertussis* infections were present in over half of the positive patients (51/84; 60.7%), and 39.3% (33/84) had associated co-infections. Of these, 28 had co-infections with another pathogen (Human Rhinovirus [*n* = 15; 17.9%], *Haemophilus influenzae* [*n* = 6; 7.1%], Human Adenovirus [*n* = 3; 3.6%], Human coronavirus NL63 [*n* = 2; 2.4%], Influenza B [*n* = 1; 1.2%], *Streptococcus pneumoniae* [*n* = 1; 1.2%], Human parainfluenza virus type 2 [*n* = 1; 1.2%], and Human bocavirus [*n* = 1; 1.2%]), while three patients had *B. pertussis* co-infections with two other respiratory pathogens (SARS-CoV2 and rhinovirus [*n* = 1; 1.2%], *Haemophilus influenzae* and rhinovirus [*n* = 1; 1.2%], *Haemophilus influenzae* and adenovirus [*n* = 1; 1.2%]).

*B. pertussis* and rhinovirus co-infections were associated with a significant increase in CRP and ferritin levels (*p* = 0.014, *p* = 0.035), with mean values of 1.3, respectively, 253.3 vs. 0.52 and 94.75 in single *B. pertussis* infections. Furthermore, a significant decrease in Sp O_2_ level (<92%) was detected (3/15, 20%) in *B. pertussis* + rhinovirus co-infections (*p* = 0.025).

Regarding the immunological status of the patients, the highest vaccination rate was observed in urban areas, reaching a percentage of 75% for those vaccinated up-to-date and 77.8% for those who were incompletely immunized. In comparison, in children in rural areas, the non-immunization rate reached 64.4% (*p* = 0.003). Among unvaccinated patients, 76.3% had interstitial lung disease, as chest X-ray images suggested (*p* = 0.008). In addition, laboratory tests revealed leukocytosis with a mean of 22.13 × 10^3^/mm^3^ (± 11.12) (*p* = 0.004), lymphocytosis—13.57 × 10^3^/mm^3^ (± 8.05) (*p* = 0.003), monocytosis—1.37 × 10^3^/mm^3^ (± 0.86) (*p* = <0.001), and thrombocytosis with a mean of 493.79 × 10^3^/mm^3^ (±128.66) (*p* = 0.044) (Table 5).

## 4. Discussion

*B. pertussis* epidemics occur every 3 to 5 years, following a cyclical pattern [19]. Romania’s last two epidemic periods were reported in 2014–2015 and 2018–2019 [20,21]. In Galati, an urban center in the southeast of Romania, the new wave of pediatric infections began in June and peaked in September 2024, unlike in other countries in Europe, which reported an earlier increase in *B. pertussis* cases. Denmark reported a surge in cases from August that peaked in the fall of 2023 [22], while in Catalonia, Italy and France, the number of infected children started to rise from January, reaching its peak in February–March, June, and July 2024, respectively [23,24,25]. The USA also reported an upward trend of pertussis in 2024, recording a peak in November [26].

In our study, most cases (53.6%) were detected in children under one year of age, of whom almost 30% were identified in 0–3 month age group. Previous studies have shown a predominance of cases in this age group, with higher rates being reported in China—from 38.4% to 41.85% in patients ≤3 months [5,27], in Tunisia—51% in children <2 months [28], in Peru—73.55% <3 months [29]. In Italy, the detection rate in this group of infants was lower, at 28.8% [30]. However, different results have been reported from northern and southern Spain, where the highest incidence was observed in the 11–15 year age group in 2023–2024 [31,32]. Considering the vulnerability of infants <2 months, the WHO recommends maternal immunization during the second or third trimester of pregnancy, as a strategy to prevent pertussis in this age group, obtaining passive immunization after maternal antibodies transfer [33,34,35,36,37]. Principi et al. demonstrated, in a recent study, that a maternal immunization of 64% reduced hospitalizations by 68%, and it reduced infections with *B. pertussis* by 78% in infants under three months [38].

In Romania, DTP1 vaccination coverage has decreased in recent years, from 89% in 2022, to 82% in 2023, and 79% in 2024 [10,39]. In addition, in a 2023 study on DTP3 vaccination coverage in Europe and Central Asia, Romania and Bosnia and Herzegovina ranked lowest, under 80% [40]. A contributing factor to the low vaccination rate in Romania is reduced access to healthcare in rural areas, as shown in our research, but also in previous studies [41], with a rate of unvaccinated children prevalent in these areas. Therefore, it is imperative to create medical facilities for patients in rural areas, and to engender a deeper preoccupation coming from doctors and nurses with a main goal of determining the rural population to vaccinate. Another cause could be the increasing hesitation of parents towards vaccination fueled by misinformation. Healthcare professionals should combat false information, becoming more involved in medical campaigns to raise awareness of the benefits of vaccination.

In our cohort, 19.1% were up to date with age-appropriate vaccination, 10.7% were incompletely vaccinated, 9.5% were not of age for vaccination, and 60.7% were not vaccinated at all. Mădălina Maria Merișescu et al. reported a higher rate of complete immunization, 21.3%, in a recent study in Bucharest [42], while Cristina Mihai et al. reported 18.4%, a similar vaccination rate to our results, in a study conducted in Constanta (Romania). In addition, the latter study shows that unvaccinated patients had higher leukocyte, lymphocyte and CRP values compared to vaccinated patients [41], results slightly different from those in our study, where CRP values according to immunological status were without statistical significance. In our analysis, unvaccinated patients were additionally associated with interstitial abnormalities on X-ray, monocytosis, and thrombocytosis. Previous studies have reported low vaccination coverage, ranging from 11% to 16.6%, in Tunisia, Denmark, and Mexico [22,43,44], but also a high percentage of 88% of age-appropriately immunized patients in Granada, Spain [31].

It is important to note that lifelong protection against pertussis cannot be obtained, and contracting the disease provides protection for a limited period, between 7 and 20 years. Pertussis immunization has been shown to decrease in protection 4–12 years from the last booster dose [45].

In the current study, no deaths were recorded. However, in 2024, five deaths were reported in Romania from whooping cough: two newborns and two infants aged 2 and 3 months, respectively, unvaccinated, and a four-month-old infant vaccinated according to age with two doses of vaccine [15]. Although *B. pertussis* affects children of all ages, unvaccinated or incompletely vaccinated infants have a more severe course [15,43,44,45,46,47]. Furthermore, in our research, infants ≤ 3 months had a higher risk of developing respiratory failure, with transfer to ICU and prolonged hospitalization, as shown in previous studies [5,30].

In the literature, co-detection of other respiratory pathogens in *B. pertussis* infection ranged from 21.4% to 76.7% [5,30,42,46,48,49]. In our study, the rate was 39.3%, with the most common pathogens detected being rhinovirus, *H.influenzae* and adenovirus. The youngest age group (0–3 months) was the most susceptible to co-infection with respiratory viruses.

The co-detection of respiratory virus in whooping cough was associated with infants ≤3 months, a different result compared to research by Wujun Jiang et al., where they showed a significant increase in co-infections at a mean age of 6 months [5]. According to previous studies, no clinical differences were reported between pertussis infection alone and co-infections [2,5,18,48,50]. Nevertheless, in our research, we observed that *B. pertussis* and HRV co-infections were correlated with decreased SpO_2_ (<92%) and increased inflammatory markers (CRP and ferritin). By contrast, Elisabetta Pandolfi et al. reported no clinical difference between *B. pertussis* infection alone and *B. pertussis* + HRV co-infection in their research [51]. In another recent study, an association was found between *B. pertussis* co-infection with *Mycoplasma pneumoniae*, *Chlamydia pneumoniae* and parainfluenza viruses [52].

The limitations of the study consist in having analyzed a single center, excluding non-hospitalized patients, and incomplete laboratory results in some cases, this being a retrospective study.

## 5. Conclusions

Pertussis is a disease that continues to persist despite vaccination efforts. As shown in our study, infants ≤3 months, who are not vaccinated, remain the most vulnerable, being susceptible to mixed co-infections and the severe course associated with leukocytosis, lymphocytosis, monocytosis, radiographic pulmonary involvement, and a long duration of hospitalization (mean 9.2 days). Furthermore, our data analysis indicated young age is a major risk factor for mixed co-infections, with the highest incidence in patients <3 months (52%), following infants up to one year (30%). Thus, preventive strategies against this disease are needed, especially in this age group, by facilitating maternal immunization during pregnancy to ensure smaximum protection of newborns. Its effectiveness has been demonstrated by reducing the risk of death by 97% [53]. In addition, continuing up-to-date postpartum vaccination should be an absolute priority in disease control.

## Figures and Tables

**Table 1:** Monthly and yearly distribution of cases.

Month	2022		2023		2024		2025		Total	
	Positive	Negative	Positive	Negative	Positive	Negative	Positive	Negative	Positive	Negative
	*n* (%)		*n* (%)		*n* (%)		*n* (%)		*n* (%)	
January	-	-	0 (0)	72	1 (1.8)	56	4 (2.7)	146	5 (1.8)	274
February	-	-	0 (0)	70	1 (1.7)	58	2 (1.5)	135	3 (1.1)	263
March	-	-	0 (0)	69	1 (1.3)	76	1 (0.8)	124	2 (0.7)	269
April	-	-	0 (0)	65	0	60	2 (1.7)	117	2 (0.8)	242
May	-	-	0 (0)	70	1 (1.7)	60	0 (0)	25	1 (0.7)	155
June	-	-	0 (0)	40	5 (8.1)	62	-	-	5 (4.9)	102
July	-	-	0 (0)	25	8 (12.7)	63	-	-	8 (9.1)	88
August	-	-	0 (0)	36	8 (13.3)	60	-	-	8 (8.3)	96
September	0 (0)	6	0 (0)	52	15 (15.8)	95	-	-	15 (9.8)	153
October	0 (0)	30	0 (0)	67	10 (10.5)	119	-	-	10 (4.6)	216
November	0 (0)	63	0 (0)	69	13 (8.4)	154	-	-	13 (4.6)	286
December	0 (0)	84	0 (0)	75	12 (6.3)	192	-	-	12 (3.4)	351

**Table 2:** General characteristics of cases.

Variables	*B. pertussis* Patients (*n* = 84)
Sex, *n* (%)	Female 47 (56%)
	Male 37 (44%)
Age (months)	
mean	29.68
median	8 (0–155)
0–3 months	25 (29.8%)
4–12 months	20 (23.8%)
13–72 months	27 (32.1%)
>72 months	12 (14.3%)
Vaccinated against pertussis, *n* (%)	25 (29.8%)
Number of DTPa-IPV-HB-Hib doses received, *n* (%)	1 dose: 11 (13.1%)
	2 doses: 3 (3.6%)
	3 doses: 8 (9.5%)
	4 doses: 3 (3.6%)
Age-appropriate immunization, *n* (%)	16 (19.1%)
Mean time elapsed from the last dose of vaccine to diagnosis (months)	31.16

**Table 3:** Clinical presentation of *B. pertussis* infection by age group.

Variables	0–3 Months	4–12 Months	13–72 Months	>72 Months	*p* Value
	*n*, %	*n*, %	*n*, %	*n*, %	
	*n* = 25	*n* = 20	*n* = 27	*n* = 12	
Sex					0.562
Female	12 (48)	13 (65)	14 (51.9)	8 (66.7)	
Male	13 (52)	7 (35)	13 (48.2)	4 (33.3)	
Symptoms					
Fever	3 (12)	3 (15)	3 (11.1)	3 (25)	0.703
Cough	25 (100)	20 (100)	26 (96.3)	12 (100)	1.000
Rhinorrhea	4 (16)	9 (45)	10 (37)	3 (25)	0.163
Dyspnea	17 (68)	5 (25)	4 (14.8)	4 (33.3)	0.001 *
Nasal obstruction	9 (36)	5 (25)	7 (25.9)	3 (33.3)	0.809
Wheezing	1 (4)	3 (15)	0 (0)	1 (8.3)	0.122
Post-cough vomiting	1 (4)	4 (20)	9 (33.3)	3 (25)	0.045 *
Apnea	5 (20)	0 (0)	1 (3.7)	1 (8.3)	0.073
Cyanosis	7 (28)	1 (5)	0 (0)	0 (0)	0.002 *
Dysphonia	1 (4)	0 (0)	1 (3.7)	1 (8.3)	0.766
Disease onset before hospitalization					0.688
≤14 days	14 (56)	14 (70)	14 (51.9)	9 (75)	
>14 days	1 (4)	3 (15)	4 (14.8)	2 (16.7)	
Physical examination Polypnea	3 (12)	0 (0)	0 (0)	0 (0)	0.112
SpO_2_ < 92%	3 (12)	0 (0)	0 (0)	0 (0)	0.165
Mixed co-infection	13 (52)	6 (30)	6 (22.2)	1 (8.3)	0.029 *
Bacterial co-infection	2 (8)	2 (10)	2 (7.4)	3 (25)	0.403
Mean LOS (SD)	9.2 (±4.5)	5.8 (±2.6)	6.3 (±2.3)	6 (±2.9)	0.022 *

* *p* < 0.05. LOS: length of stay.

**Table 4:** Complications in *B. pertussis* patients.

Complications	0–3 Months	4–12 Months	13–72 Months	>72 Months	*p* Value
	*n*, %	*n*, %	*n*, %	*n*, %	
	*n* = 25	*n* = 20	*n* = 27	*n* = 12	
Pneumonia	21 (84)	14 (70)	23 (85.2)	10 (83.3)	0.584
Respiratory failure	5 (20)	0 (0)	1 (3.7)	0 (0)	0.035 *
Single infection	11 (44)	13 (65)	19 (70.4)	8 (66.7)	0.229
Co-infections	14 (56)	7 (30)	8 (29.6)	4 (33.3)	
*B. pertussis* + HRV	8 (32)	4 (20)	4 (14.8)	1 (8.3)	0.528
*B. pertussis* + *H. influenzae*	2 (8)	2 (10)	2 (7.4)	2 (16.7)	0.809
*B. pertussis* + HAdV	1 (4)	1 (5)	2 (7.4)	0 (0)	1.000
*B. pertussis* + HCoV NL63	2 (8)	0 (0)	0 (0)	0 (0)	0.511
Transfer to ICU	8 (32)	0 (0)	0 (0)	0 (0)	<0.001 *
Supplemental Oxygen					
Oxygen therapy	3 (12)	0 (0)	1 (3.7)	0 (0)	0.362
Mechanical ventilation	1 (4)	0 (0)	0 (0)	0 (0)	0.679

* *p* < 0.05. ICU: Intensive care unit; HRV: Human Rhinovirus; HAdV: Human Adenovirus; HCoV NL63: Human coronavirus NL63.

**Table 5:** Epidemiological, clinical and paraclinical data of *B. pertussis* cases according to immunological status.

Variables	Vaccinated	Incompletely	Unvaccinated	*p* Value
	*n* = 16	Vaccinated, *n* = 9	*n* = 59	
Sex, *n* %				0.144
Female	12 (75)	6 (66.7)	29 (49.2)	
Male	4 (25)	3 (33.3)	30 (50.9)	
Area, *n* %				0.003 *
Urban	12 (75)	7 (77.8)	21 (35.6)	
Rural	4 (25)	2 (22.2)	38 (64.4)	
Mixed co-infections, *n* %	4 (25)	0 (0)	22 (37.3)	0.065
Bacterial co-infections, *n* %	2 (12.5)	3 (22.2)	4 (6.8)	0.051
Prolonged LOS > 7 days, *n* %	5 (31.3)	3 (33.3)	22 (37.3)	0.894
Transfer to ICU, *n* %	0 (0)	0 (0)	8 (13.6)	0.224
Oxygen therapy, *n* %	0 (0)	0 (0)	4 (6.8)	0.73
Chest X-ray, *n* %				0.008 *
Interstitial	9 (56.3)	6 (66.7)	45 (76.3)	
Condensation	0 (0)	1 (11.1)	0 (0)	
Opacity	1 (6.3)	0 (0)	0 (0)	
Normal	0 (0)	1 (11.1)	0 (0)	
SpO_2_ < 92%, *n* %	0 (0)	0 (0)	3 (5.1)	0.69
Leukocytes, mean ± SD	14.41 (±5.59)	14.28 (±4.54)	22.13 (±11.12)	0.004 *
Neutrophils, mean ± SD	4.99 (±2.23)	5.59 (±4.01)	6.67 (±4.24)	0.353
Lymphocytes, mean ± SD	8.06 (±5.27)	7.26 (±2.38)	13.57 (±8.05)	0.003 *
Monocytes, mean ± SD	0.71 (±0.33)	0.83 (±0.41)	1.37 (±0.86)	<0.001 *
CRP, mean ± SD	0.59 (±0.36)	0.7 (±0.56)	0.76 (±0.99)	0.509
PLT, mean ± SD	460.78 (±133.99)	391.44 (±80.93)	493.79 (±128.66)	0.044 *

* *p* < 0.05. LOS: length of stay; ICU: intensive care unit.

## Data Availability

The raw data supporting the conclusions of this article will be made available by the authors on request.

## Abbreviations

The following abbreviations are used in this manuscript:

ICU	Intensive care unit
CRP	C-reactive protein
χ^2^	Chi-squared test
SpO_2_	Oxygen saturation level measured by a pulse oximeter
LOS	Length of stay
PLT	Platelet count
